# Supplementary figures and images for: Intracellular polarization of RNAs and proteins in the human small intestinal epithelium
Source: PLoS Biol. 2024 Dec 2;22(12):e3002942. doi: 10.1371/journal.pbio.3002942 (PMC11637431; doi:10.1371/journal.pbio.3002942)

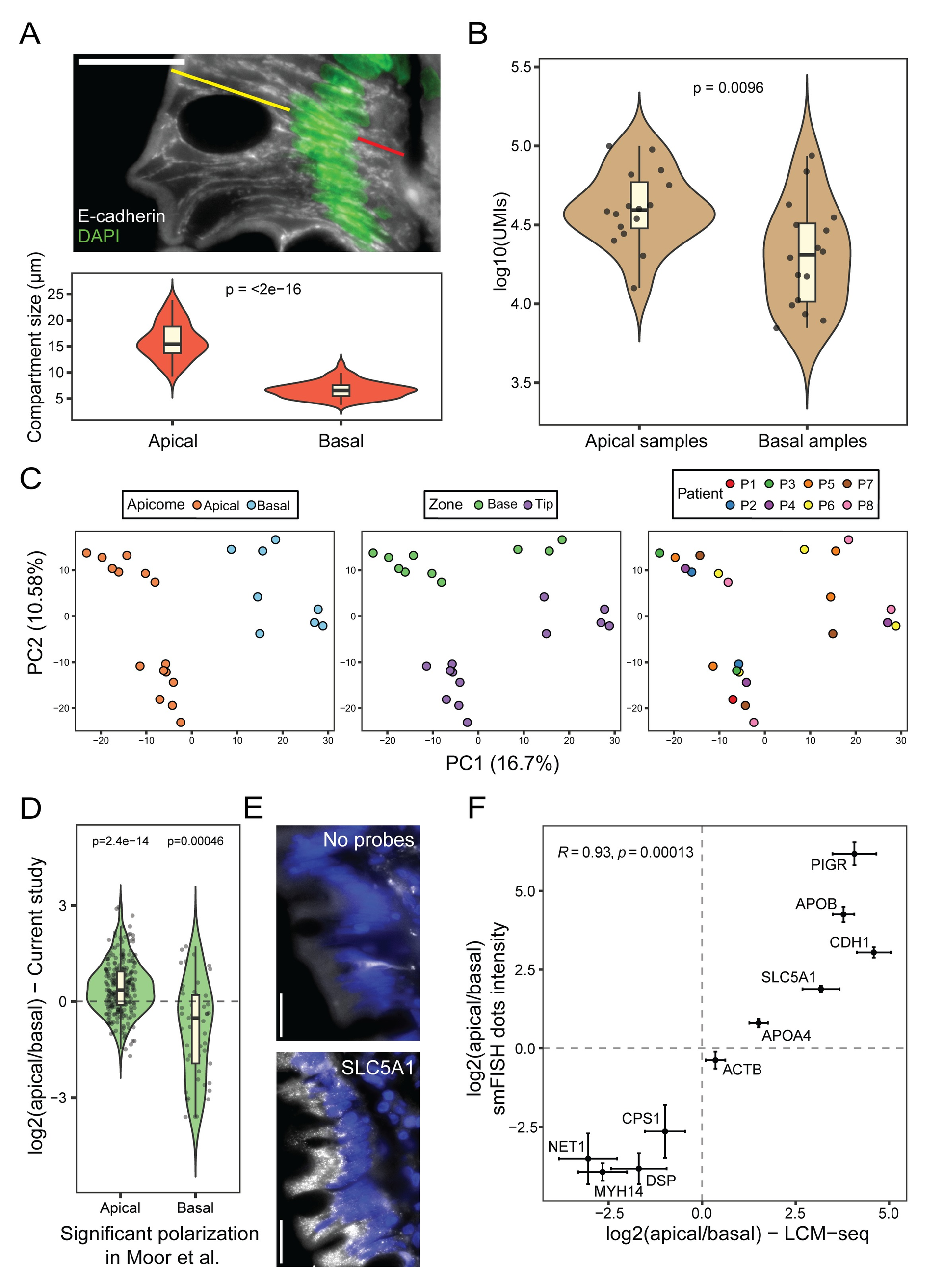

Supplement: S1 Fig — (A) Top–Immuno-fluorescence staining of E-cadherin, used for measurement of apical (yellow) and basal (red) sides of the epithelial cells. Scale bar 20 μm. Bottom–quantification of measurements from 8 patients, 3 FOV per patient, 3 measurements per FOV. Boxplots show the medians and 25–75 percentiles, p-value is Wilcoxon rank-sum test. Data is available in S2 Table and S1 Data. (B) Number of unique molecules identifiers (UMIs) across apical and basal samples of LCM RNA-seq of human proximal jejunums. Boxplots show the medians and 25–75 percentiles, p-value is Wilcoxon rank-sum test. Data is available in S3 Table and S1 Data. (C) Principal component analysis (PCA) of human LCM RNA-seq, samples colored by cellular compartment (left), by villus zone (mid) or by patient (right). Only samples with more than 20,000 UMIs and genes with mean normalized expressions of more than 10−4 are included. Data is available in S3 Table and S1 Data. (D) log2(apical/basal) from LCM RNA-seq of 5 mice. Apical/basal classification based on previously published data of mouse apicome [3]. Shown are genes that are significantly polarized in Moor and colleagues data set (q-value < 0.1). Horizontal bars are medians, boxes delineate the 25–75 percentiles. Data is available in S3 Table and S1 Data. (E) smFISH staining of SLC5A1 and a staining without a probe. Scale bar is 20 μm. (F) Spearman correlation between the log2(apical/basal) in LCM RNA-seq and quantification of dot intensity of smFISH. In smFISH, intensity of dots (normalized to area), from multiple fields of view, from 2 to 3 patients were quantified. Error bars are standard errors of the means of the log2(apical/basal) from all samples or measurements. SmFISH quantification and RNA-seq data are available in S2 and S3 Tables, respectively, and S1 Data. (TIF) [file pbio.3002942.s001.tif]

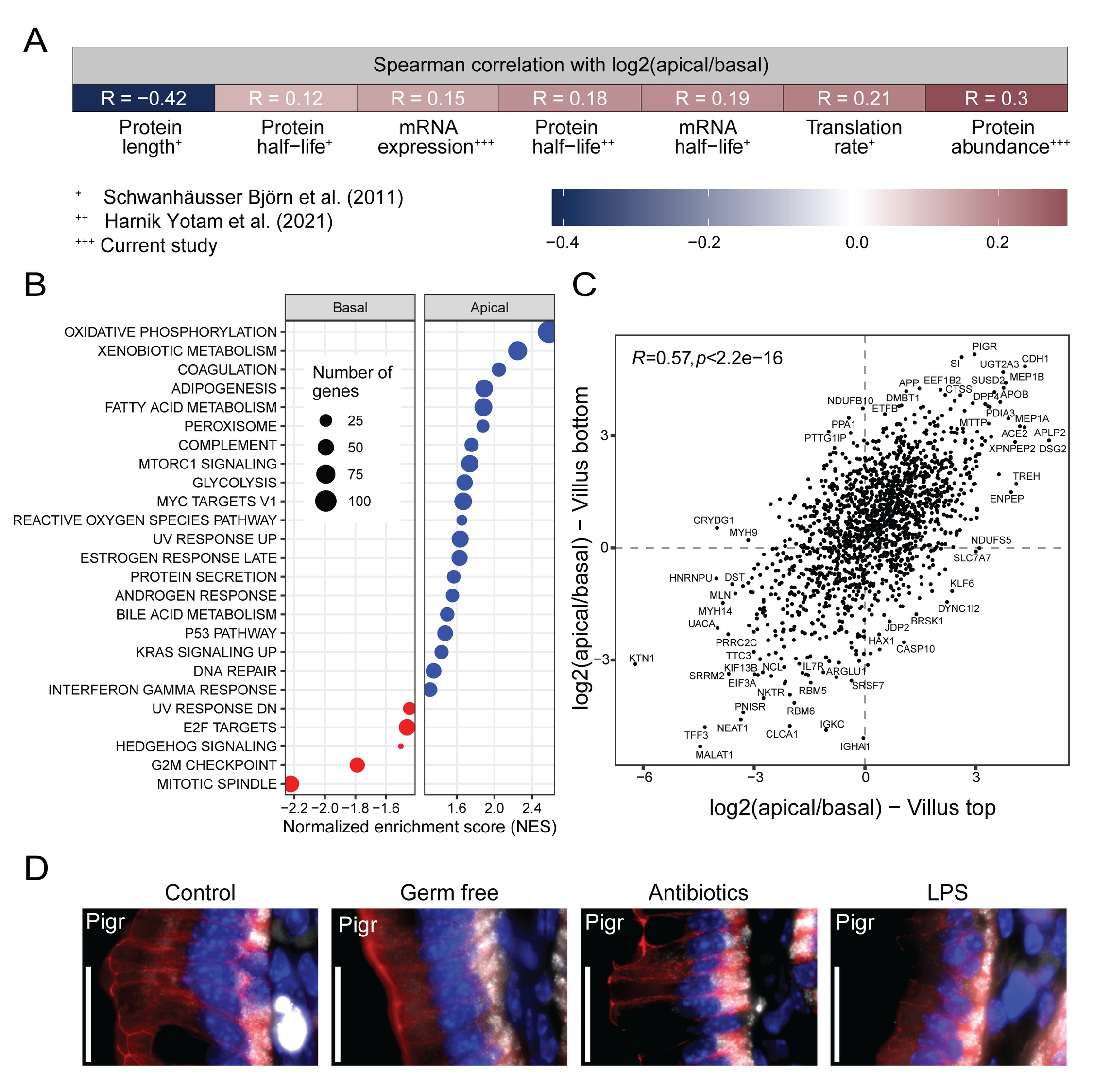

Supplement: S2 Fig — (A) Spearman correlation of log2(apical/basal) ratios from LCM RNA-seq and different gene parameters, taken from Schwanhäusser and colleagues [26] and Harnik and colleagues [25]. mRNA expression and protein abundance values are the averages of normalized expression data from the current study. (B) Gene set enrichment analysis (GSEA) results based on log2(apical/basal) of LCM RNA-seq. Only pathways with q-value < 0.1 are shown. Data is available in S3 Table and S1 Data. (C) Spearman correlation between the log2(apical/basal) in human data, in bottom and tip of villi. Only genes with normalized expression >10−4 are included. Data is available in S3 Table and S1 Data. (D) smFISH staining of Pigr in the jejunums of mice in different conditions. In red–E-cadherin immunofluorescence, in blue–DAPI. Scale bar is 20 μm. (TIF) [file pbio.3002942.s002.tif]

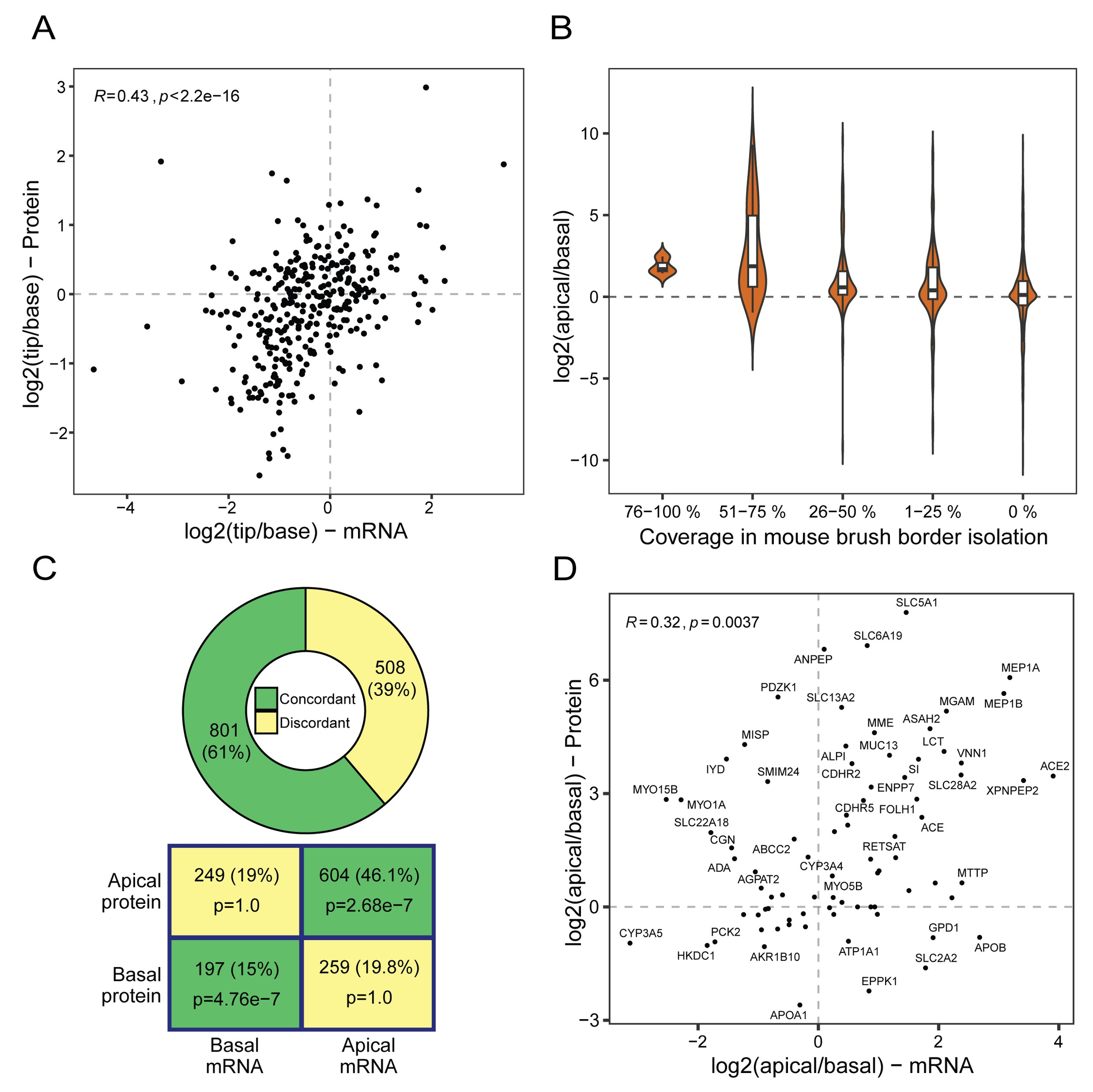

Supplement: S3 Fig — (A) Spearman correlation of the log2(tip of villi/base of villi), of LCM RNA-seq and proteomics. Only genes with normalized expression in both data sets >10−4 are included. RNA-seq and proteomics data are available in S3 and S4 Table, respectively. (B) Protein log2(apical/basal), where proteins are classified based on the coverage in mouse brush border isolation from McConnell and colleagues [6]. Horizontal bars are medians, boxes delineate the 25–75 percentiles. (C) Bottom–number of genes in each state, depending on the polarization of the mRNA and the corresponding protein. P-values are based on hypergeometric tests. Top–distribution of concordant and discordant genes, colors correspond to the colors in the bottom panel. Data is available in S4 Table and S1 Data. (D) Spearman correlation between the log2(apical/basal) of mRNAs and the corresponding log2(apical/basal) of proteins, Only epithelial specific genes are included (Methods). RNA-seq and proteomics data are available in S3 and S4 Tables, respectively, and in S1 Data. (TIF) [file pbio.3002942.s003.tif]

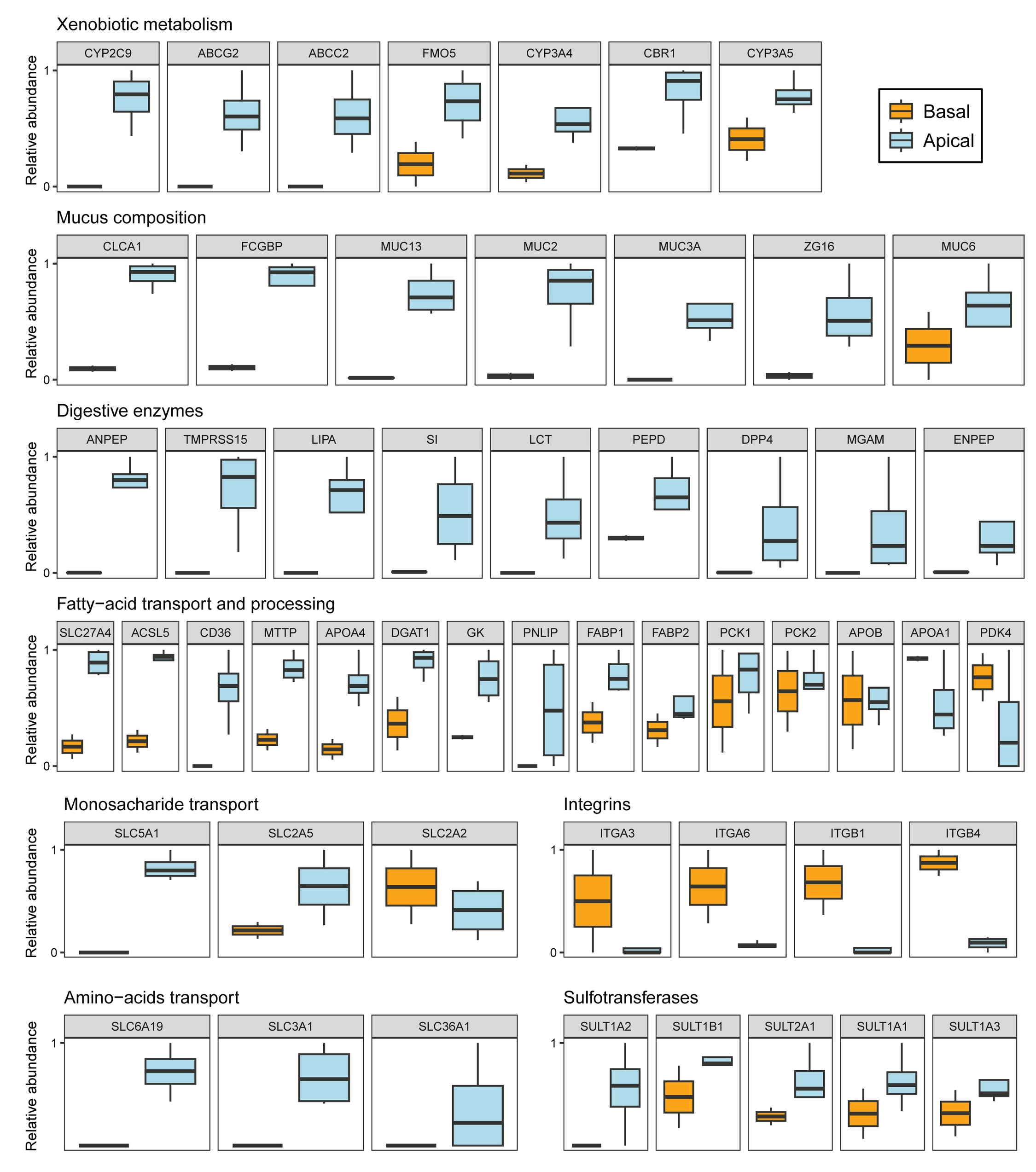

Supplement: S4 Fig — Horizontal bars are medians, boxes delineate the 25–75 percentiles. Data is available in S4 Table and S1 Data. (TIF) [file pbio.3002942.s004.tif]

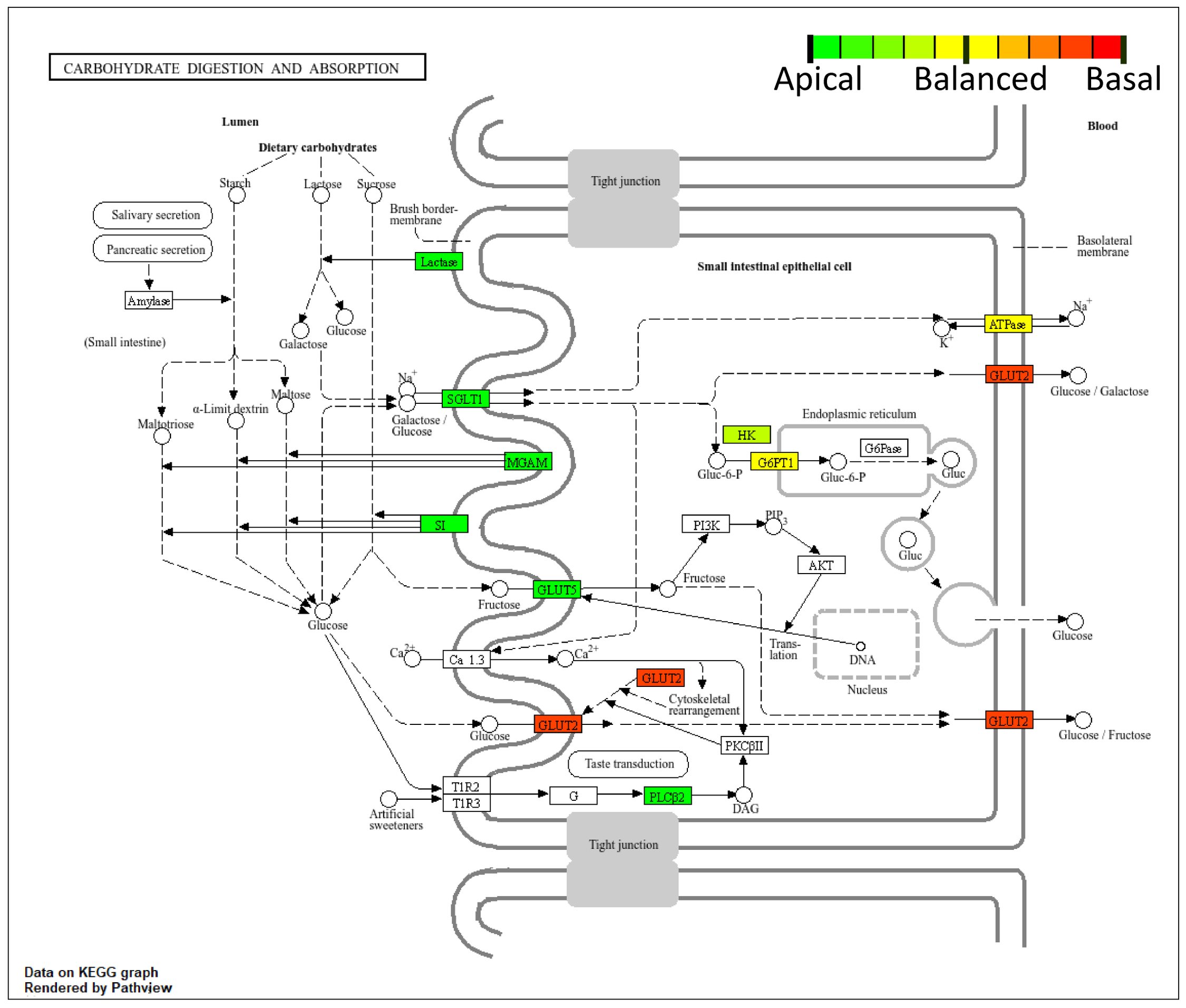

Supplement: S5 Fig — Color is determined by the log2(apical/basal) of LCM-proteomics, where all values above 1 are rounded to 1, and all values below −1 are rounded to −1. Visualized with Pathview [46]. (TIF) [file pbio.3002942.s005.tif]

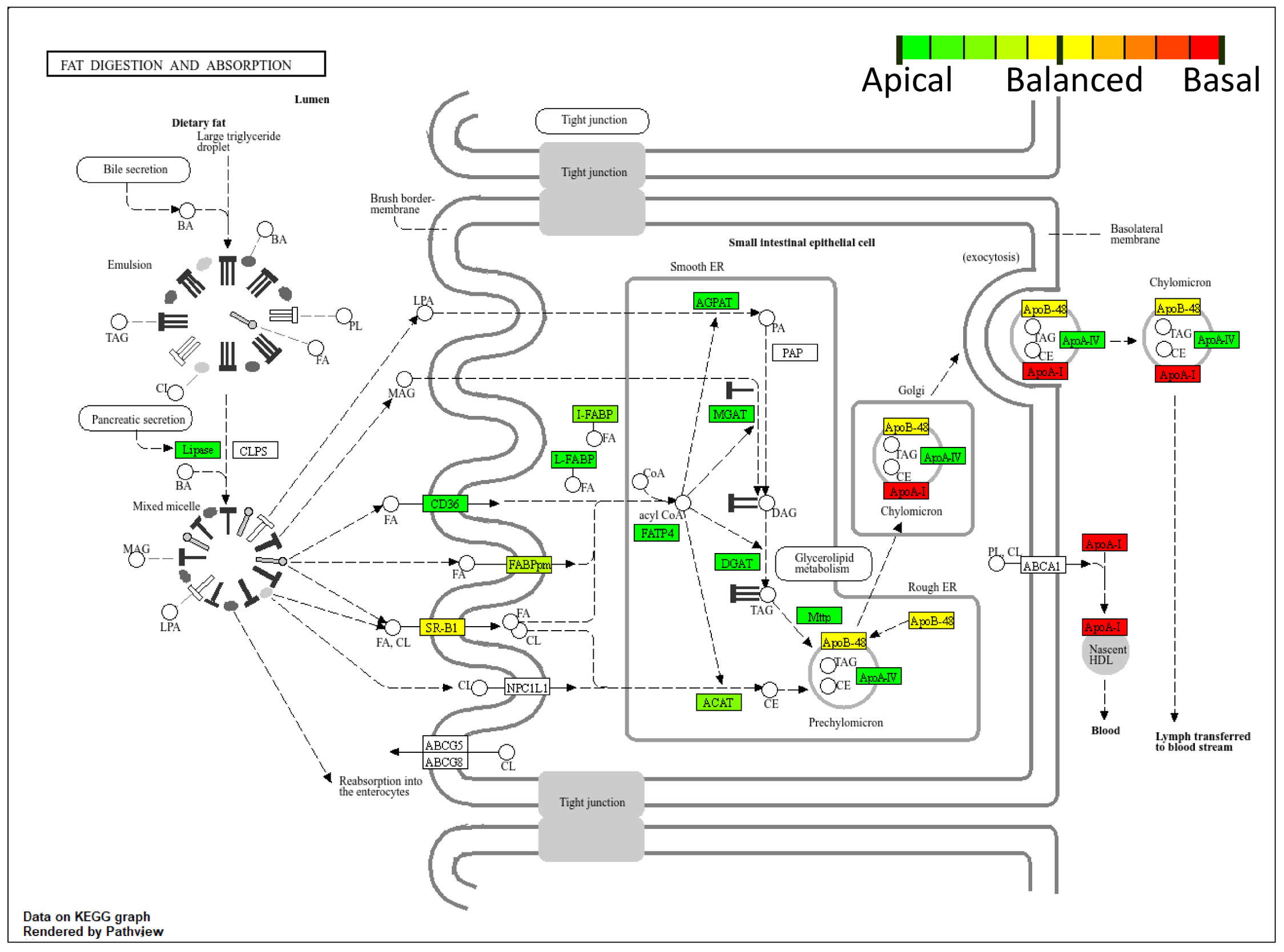

Supplement: S6 Fig — Color is determined by the log2(apical/basal) of LCM-proteomics, where all values above 1 are rounded to 1, and all values below −1 are rounded to −1. Visualized with Pathview [46]. (TIF) [file pbio.3002942.s006.tif]

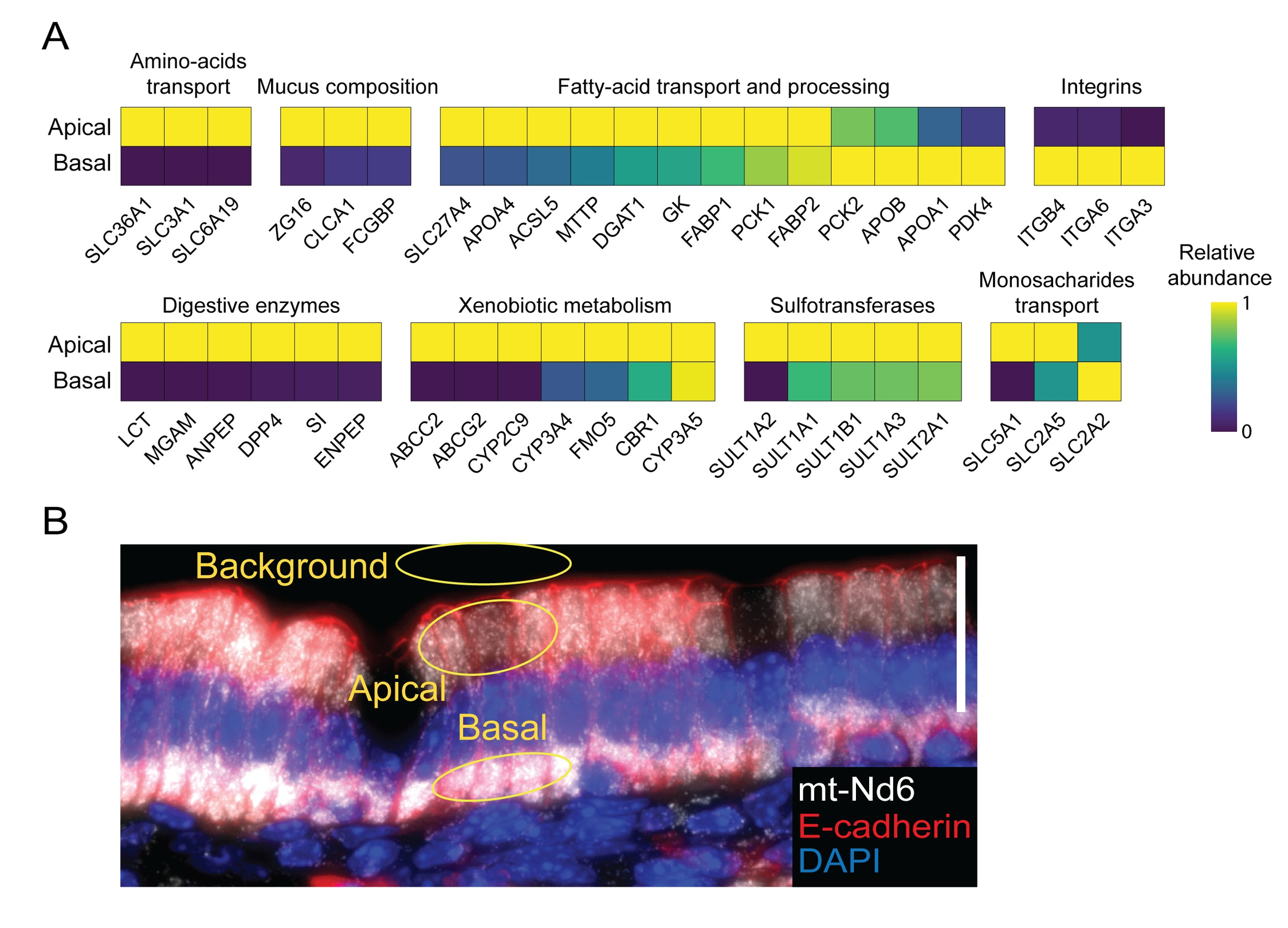

Supplement: S7 Fig — (A) Max-normalized abundance of cytoplasmic proteins involved in nutrient processing and absorption on the apical and basal sides of cytoplasmic proteins from LCM-proteomics data. The data is internally normalized after filtering out nuclear and ECM proteins (Methods). (B) smFISH image of mt-Nd6 in mouse jejunum, as example of segmentation and quantification of the intensity. Scale bar 20 μm. (TIF) [file pbio.3002942.s007.tif]
