# Supplementary material for: Intracellular polarization of RNAs and proteins in the human small intestinal epithelium
Source: PLoS Biol. 2024 Dec 2;22(12):e3002942. doi: 10.1371/journal.pbio.3002942 (PMC11637431; doi:10.1371/journal.pbio.3002942)
Supplement: Attachment — Submitted filename: Response to Reviewers.pdf [file pbio.3002942.s014.pdf]

Dear Ines,

We thank you and the reviewers for your excellent and constructive comments. We have now addressed all the comments with extensive new experiments and analyses. Specifically:

- We have performed quantification of smFISH and correlated the results to the LCM-RNAseq results. This strongly validated the RNA-seq results.
- We have performed additional experiments in which we assayed Pigr polarization in germ-free mice, in mice that have been treated with wide-spectrum antibiotics and in mice injected with LPS. We show that Pigr polarization remains invariable under these diverse perturbations, strengthening our conclusion that the difference in polarization of this gene is specie- dependent.
- We have analyzed the correlation of the apical bias of mRNAs and various parameters such as mRNA level, stability and protein length. Additionally, we have performed functional gene-set enrichment analysis based on the genes' apical biases.
- We have reanalyzed the proteomics analysis while excluding nuclear-localized proteins and ECM proteins. We show that the epithelium exhibits broad protein intra-cellular polarization even after removal of these gene sets.
- We have added new figures and analyses to thoroughly address all additional points raised by the reviewers.

We believe that these new experiments and analyses have significantly strengthened our work. Below we highlight our responses to each of the reviewers' comments. We marked the new text with underline in the manuscript sections.

Reviewers' comments

Academic Editor:

The Academic Editor would like you to comment on the difference in the subcellular distribution of PIGR, which is apical in humans, but not in mice. Since the expression is regulated by cytokines in response to pathogenic stimuli, one may wonder whether this change in protein localization is state-dependent, rather than species-specific.

We thank the Academic editor for his valuable comment. In our human data, PIGR mRNA consistently shows clear apical polarization across all patients and similarly, clear basal polarization in all mice (*Fig. R1, see also Fig. 2D and Supplementary Fig. 1F*).

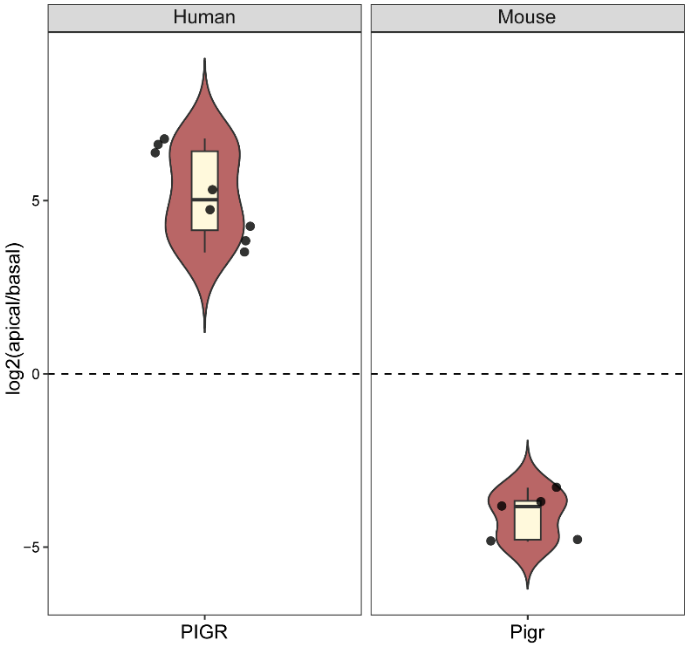

Fig. R1: ***Ratio between apical and basal concentrations of the mRNA of PIGR in human (left) and mouse (right). Each dot is an individual, horizontal bars are medians, boxes delineate the 25-75 percentiles.***

To investigate whether the polarization of Pigr might depend on inflammation level, we have now assayed Pigr polarization in a range of conditions - germ-free mice and antibiotic treated mice, emulating low inflammation states, and Lipopolysaccharide (LPS)-injected mice, which mimic bacterial infection after intestinal barrier breach (https://www.ncbi.nlm.nih.gov/pmc/articles/PMC6101093/). We found that Pigr intra-cellular polarization remained basal in all conditions (new Fig. S2D). This suggests that the difference in polarization is species-dependent, rather than inflammation-state dependent. We now present these new results in the text on page 4:

"Only 5 genes changed their polarization between the two species and were also significantly polarized in at least one of them – PIGR, SLC5A1, RACK1, MGAM, CDHR5 (Fig. 2C). We validated two of these genes with smFISH – PIGR was significantly apical in humans yet significantly basal in mice, whereas SLC5A1 was significantly apical in humans yet non-polarized in mice. We also validated APOB, which shows apical polarization in both species (Fig. 2D). As inflammation has been shown to upregulate Pigr expression via cytokines (4), we checked whether the polarization of Pigr mRNA changes in germ-free mice, antibiotic treated mice and lipopolysaccharides (LPS) injected mice. We found that the polarization remained basal in all conditions (Fig. S2D)."

And in the new Fig. S2D:

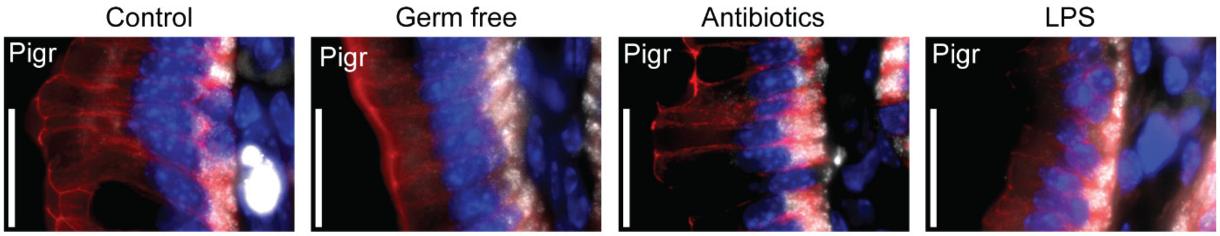

Fig. S2D: smFISH staining of Pigr in the jejunums of mice in different conditions. In red – E-cadherin immunofluorescence, in blue – DAPI. Scale bar is 20μm.

And discuss the results on page 10:

"A notable example of a gene that showed different polarization between both species was PIGR, which showed basal polarization in mouse and apical polarization in human. The localization of Pigr in mice remained constant in various conditions that modulate immune status, suggesting that the change is specie-dependent rather than state-dependent."

Rev. 1:

The manuscript "Intra-cellular polarization of RNAs and proteins in the human small intestinal epithelium" deciphers and compares the spatial distribution of mRNA molecules and proteins in mouse and human intestinal epithelial cells along the villus axis. By utilizing laser-capture microdissection, the study generates high-throughput transcriptome and proteome data from intracellular apical and basal compartments, creating a novel apical-basal polarization profile (apicome) for the human intestinal epithelium. The authors demonstrate clear polarization of mRNA molecules and proteins in the human intestinal epithelial cells, similar to patterns observed in mice. This study also highlights significant differences in polarization patterns between the two species, including a lack of ribosomal protein polarization in humans, suggesting that the previously identified RNA polarization-dependent machinery regulating translation rate in mice does not exist in humans. The characterized apicome of proteins involved in nutrient transport and metabolic processes aligns with their known in vivo functions, underscoring the significance of these findings for understanding human epithelial cell functions. Additionally, the comparative study is valuable for further investigation on species-specific cellular mechanisms and their evolutionary implications. While this manuscript is well written, it is an incremental advance in our understanding of intestinal biology. Additional analyses would help strengthen the significance.

1. The manuscript demonstrates RNA and protein polarization within mouse and human intestinal epithelial cells but did not perform any functional validation of physiological relevance. For example, it would be interesting to investigate the correlation between protein turnover rates and RNA polarization, as well as between mRNA stabilization and localization. (Why is RNA polarization required when ribosomes are evenly distributed in human epithelial cells?) Are there certain classes of proteins/genes that have localized mRNA/protein?

We thank the reviewer for the excellent idea of investigating correlation of the apicome to half-lives of mRNAs and proteins. We have now performed this analysis, as well as few additional correlations. We describe the results in page 4:

"The apical bias of mRNAs had a negative correlation with protein lengths, and positive correlation with mRNA expression levels and stabilities, as well as with translation rates, protein abundances and stabilities (Fig. S2A). Therefore, apical mRNAs seem to be shorter, more stable and more efficiently translated. "

We show the results in Fig. S2A:

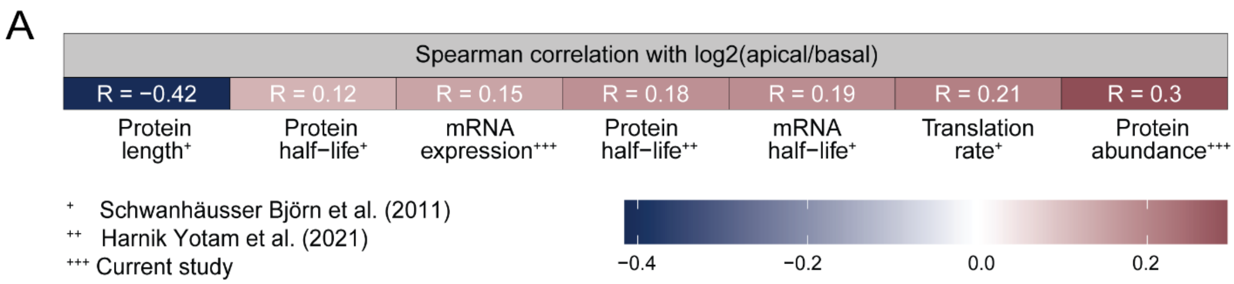

Fig. S2A: <u>(A) Spearman correlation of log2(apical/basal) ratios from LCM RNA-seq and different gene parameters, taken from Schwanhäusser B. et al. (2011) (26) and Harnik Y. et al. (2021) (25). mRNA expression and protein abundance values are the averages of normalized expression data from the current study.</u>

And discussed the results in page 11:

"We found that the distribution of the ribosomes across the apical/basal compartments in humans is uniform (Fig. 4B-C,F), in contrast to mice. Despite the uniform distribution, we found a correlation between the polarization of mRNAs and their translation rates (Fig. S2A). This discrepancy could be explained by the presence of shorter, more abundant mRNAs, in the apical side – both of which are closely linked to translation rates (25–27). Additionally, intracellular localization of mRNAs and proteins, including ribosome components, might be influenced by feeding status, a phenomenon that would be interesting to explore in future studies. "

We have now also performed gene-set enrichment analysis on the genes ranked by their mRNA apical/basal polarization, the results of which are presented on page 4:

"Additionally, the apical genes were enriched in metabolic functions such as oxidative phosphorylation, glycolysis and fatty acid metabolism, while the basal genes were enriched in mitotic spindle pathway genes (Fig. S2B, Supplementary Table 3). "

And in Fig S2B:

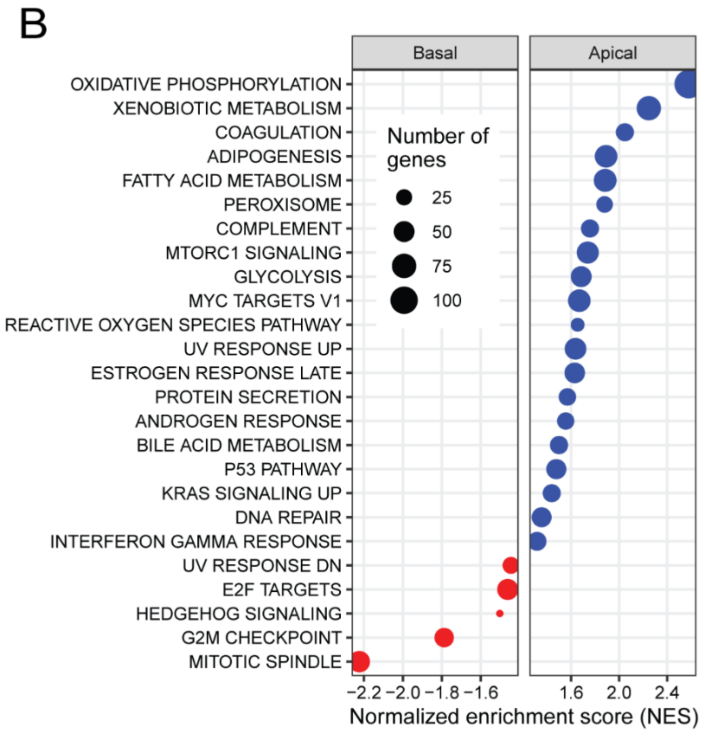

Fig. S2B: Gene set enrichment analysis (GSEA) results based on log2(apical/basal) of LCM-RNAseq. Only pathways with q-value < 0.1 are shown.

Performing GSEA on the proteomics data revealed no significant pathways, but we do show the polarization of some manually curated pathways that are enriched in the apical and basal compartments, as well as pathways with a streamlined process, shown in Fig. 4A:

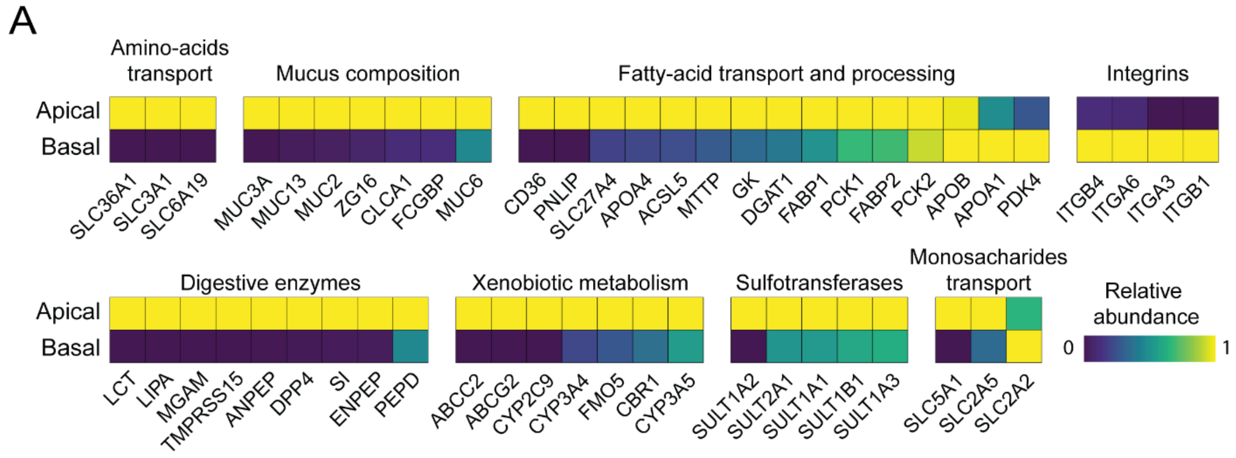

Fig. 4A: Max-normalized abundance of proteins involved in nutrient processing and absorption on the apical and basal sides from LCM-proteomics data.

2. The authors conclude that "proteins and mRNAs in human enterocytes are generally localized in their apical/basal cellular compartments". However, the Spearman correlations in fig.3C and fig.S2B (R=0.13 and R=0.32) are not strong enough to support this conclusion. It would also be helpful to show the percentage of polarized RNAs that share the same localization as their encoded proteins.

We have changed the text to reflect the correlation:

"This suggests that <u>*some*</u> *proteins and mRNAs in human enterocytes are co-localized in their apical/basal cellular compartments".*

Additionally, we have added a supplementary Fig. S3C that shows the number of concordant and discordant genes:

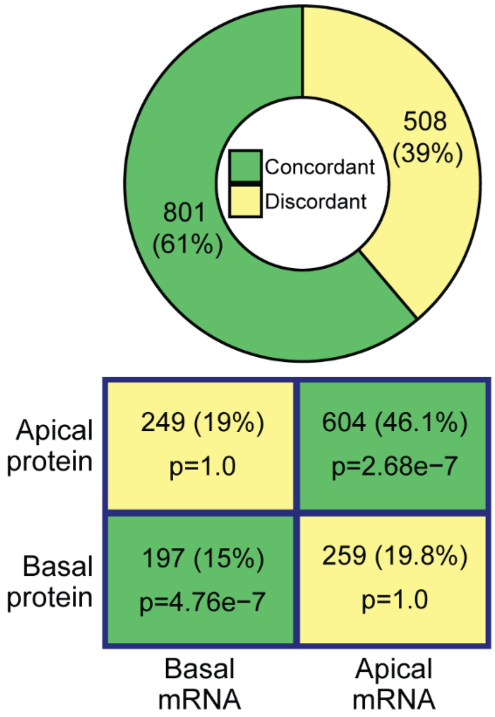

Fig. S3C: Bottom – number of genes in each state, depending on the polarization of the mRNA and the corresponding protein. P-values are based on hypergeometric tests. Top – distribution of concordant and discordant genes, colors correspond to the colors in the bottom panel.

We describe this analysis on page 6:

"*We found a weak, yet significant positive correlation between the apical bias of mRNAs and their matching proteins (Spearman's R=0.13, p=7.8 × 10-6. Fig. 3C), with the majority of genes exhibiting co-localization of proteins and mRNAs (61%, Fig. S3C)."*

3. The expression levels of certain proteins are different between the villus tip and bottom. It would be interesting to examine the differences in RNA polarization at various villus regions for those proteins to find possible relationship between translation efficiency and RNA localization.

The reviewer raises an interesting point, namely that changes in intra-cellular RNA polarization could impact translation rates. This is particularly relevant in mouse, where ribosomes are more abundant in the apical sides, and where apical polarization has been shown to correlate with translation efficiency. We have examined whether there are mRNAs that change the polarization along the villus axis in both human and mouse with the aim of correlating with potential changes in translation rates. Notably, we found no genes that change their polarization significantly – implying that mRNA polarization is constant along the villus axis:

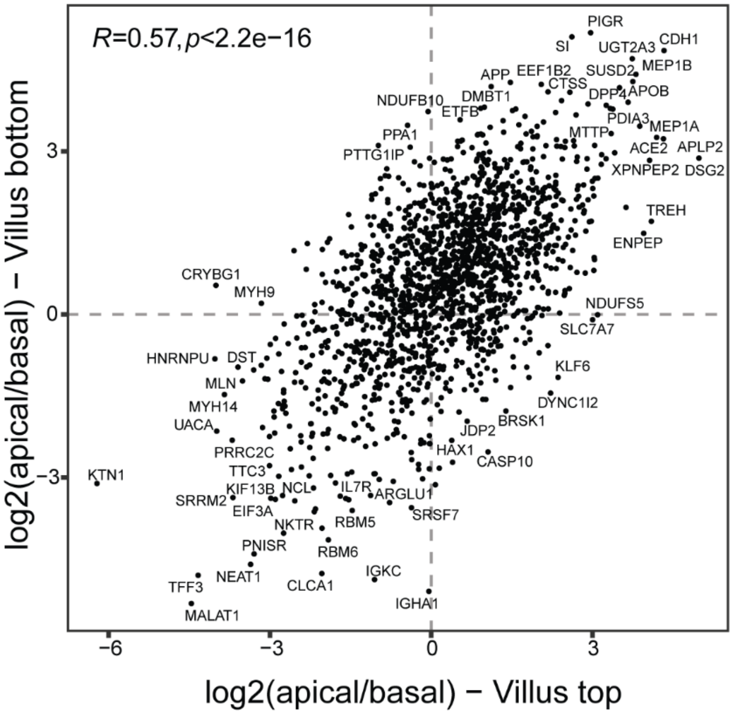

Fig. S2C: Spearman correlation between the log2(apical/basal) in human data, in bottom and tip of villi. Only genes with normalized expression > 10^{-4} are included.

We present the results of this point on page 4:

"None of the highly expressed genes changed their polarization between the base and tip of the villi in either human or mouse (Fig. S2C, no genes had q-value lower than 0.5). Our data therefore exposes mRNA polarization in the human intestinal epithelium, that seems to be invariable in distinct villi zones."

And in the discussion, on page 11:

"*In our study, we did not identify zonal changes in polarization patterns in either humans or mice, suggesting that changes in polarization may not be the mechanism through which zonal modulation of translation rates is achieved. In line with this finding, a study that reconstructed villus zonation profiles of enterocyte mRNAs and proteins in mouse suggested that translation rates are largely constant along the villus axis (25).*"

4. The statistical analysis in fig.4E-G requires more detailed explanation.

We have now clarified the caption of the figure:

"(E) The log2(apical/basal) of mitochondrially-encoded genes with normalized expression above 10^{-4} in both human and mouse, based on RNA-seq. P-values are based on the paired Wilcoxon signed rank test. Horizontal bars are medians, boxes delineate the 25-75 percentiles. (F-G) Fluorescence intensity of smFISH stainings in the apical and basal sides of ribosomal (F) and mitochondrial (G) RNA. Each measurement consists of the median intensity across 3-5 epithelial cells from 8 patients and 3 mice. P-values are based on the paired Wilcoxon signed rank test for each patient/mouse, combined by the Fisher method for multiple p-values (Methods). Horizontal bars are medians, boxes delineate the 25-75 percentiles."

And in the methods on page 18:

"Segments from the apical and basal sides of 3-5 epithelial cells from spatially distant villi were manually segmented on nuclear and membrane staining using imageJ (37) for each of the eight patients and of three mice (Fig. S2C). The median intensity for mitochondrial and ribosomal stainings were quantified in both sides, and the corresponding median intensity of the background was subtracted. Ratio of apical/basal background-subtracted intensities was calculated, and two signed-rank tests were performed on each patient, one test with left-tail and one with right-tail hypothesis. The minimal P-value of both tests was combined across the eight patients and across the three mice using Fisher's method."

5. In fig.S1A, the clustering in the principal component analysis of human LCM-RNAseq data is not clear and distinct. Only apical compartment samples show robust clustering.

We have now revised our PCA analysis to include only samples with sufficiently high UMI counts (>20,000), and highly expressed genes (mean normalized expression across samples >10^{-4}). This analysis of higher quality samples and genes more clearly reveals the separation between tip-base and apical-basal:

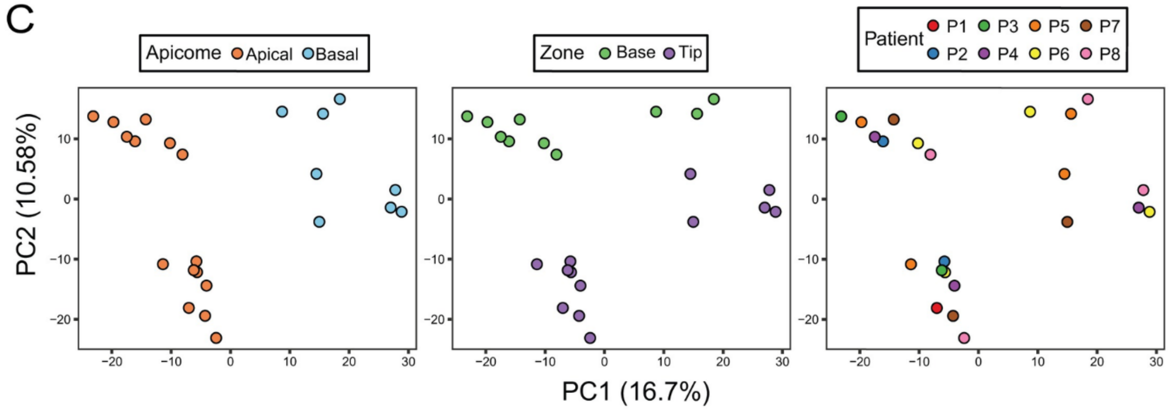

Fig. S1C: Principal component analysis (PCA) of human LCM-RNAseq, samples colored by cellular compartments (left), by villus zones (mid) or by patients (right). Only samples with more than 20,000 UMIs and genes with mean normalized expressions of more than 10^{-4} are included.

Rev. 2:

This is one of a number of high impact studies from the Itzkovitz laboratory and studies apical and basolateral polarity of mRNA, mitochondria and proteins comparing human and mouse. There are several concerns with the study although except for the first point they are minor:

1. The methodology allows both apical and basolateral separation as well as villus tip and villus base separation. However, striking is lack of analysis of the villus tip and villus base domain separation in human that was so well done in the mouse; with the hugely important difference between mRNA and protein zonation. It is reasonable to ask that this analysis be added to the manuscript, although this would be far less in detail than was provided for the mouse, it would still be impactful.

We agree with the reviewer that the zonation of genes along the villus axis is interesting and have now added this analysis in the new supplementary figure S3A, showing that zonated changes in mRNA and proteins are generally concordant in human:

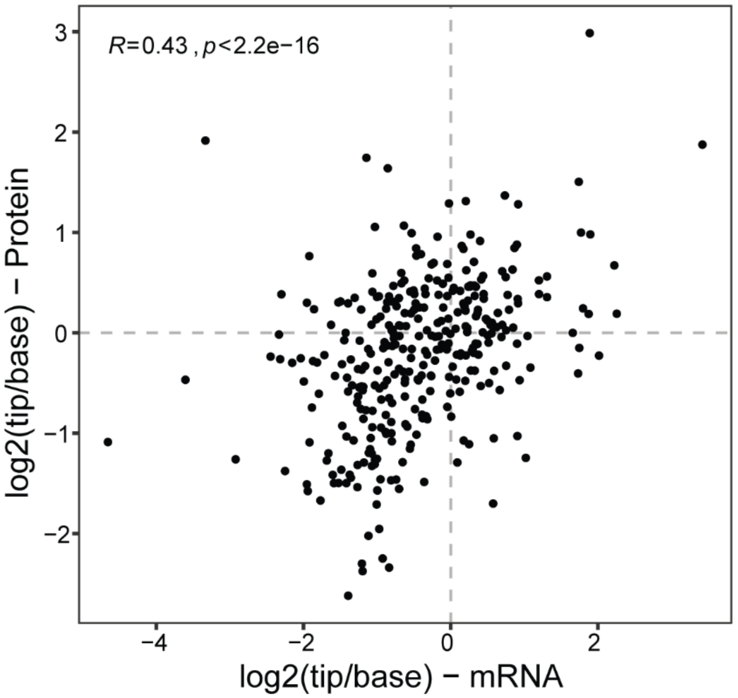

Fig. S3A: Spearman correlation of the log2(tip of villi/base of villi), of LCM-RNAseq and proteomics. Only genes with normalized expression in both datasets > 10^{-4} are included.

This is described on page 6:

"*<u>We found a significant correlation between the zonated changes in mRNAs and proteins along the villus axis (Fig. S3A, Methods). This positive correlation is in line with recent analysis of whole epithelial segments (4).</u>*"

Importantly, the issue of zonation along the villus axis in human has been thoroughly investigated in a recent publication from our lab (Fig. R2, Extended data figure 9g in

https://www.nature.com/articles/s41586-024-07793-3), and we therefore do not go into depth in discussing these specific results in our current manuscript, which focuses on intra-cellular polarization.

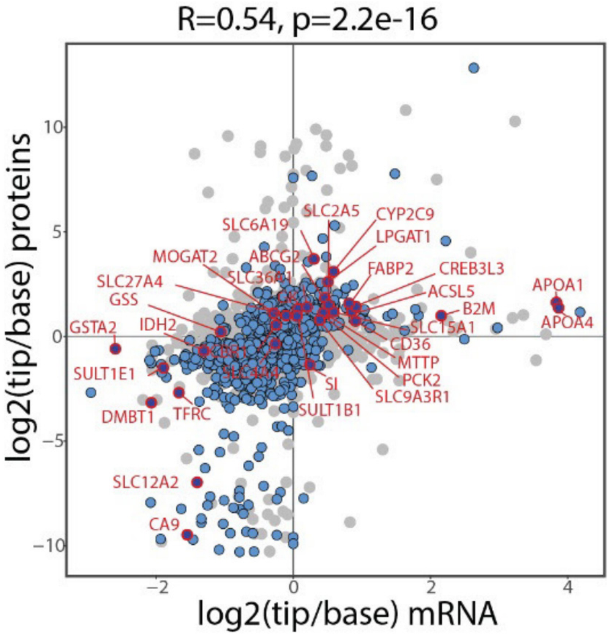

Fig. R2: Spearman correlation (R = 0.54, two-sided p = 2.2 × 10^{-16}) of the villus height bias of mRNA (based on spatial transcriptomics) and the corresponding proteins (based on LCM proteomics). Shown are genes with sum-normalized expression above 10^{-6} (in either RNA or protein). Spearman R is calculated on significantly polarized genes between the LCM samples, colored by blue (exact Wilcox rank-sum with BH correction, Q-val<0.3). Circled in red – examples of genes mentioned throughout the study. Figure taken from Harnik et al. (https://www.nature.com/articles/s41586-024-07793-3).

2. Add some comment why think chemotherapy in human patients not affecting result.

Our cohort of eight patients included 4 patients who underwent chemotherapy and 4 chemotherapy-naïve patients. In the PCA we performed we can see that the samples cluster by the apical/basal polarization and villus zonal origin, rather than by chemotherapy status, which suggests that chemotherapy does not significantly affect intra-cellular polarization (Fig. R3):

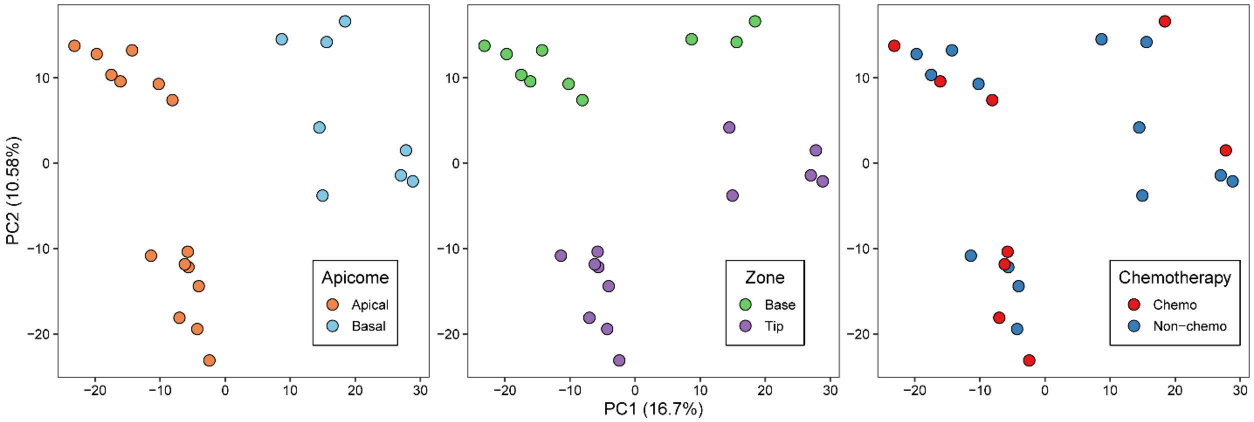

Fig. R3: Principal component analysis (PCA) of human LCM-RNAseq, samples colored by cellular compartment (left), by villi zone (mid) or by chemotherapy status (right). Only samples with more than 20,000 UMIs and genes with normalized expressions of more than 10^{-4} are included.

To further map the effect of chemotherapy on the apical/basal polarization, we have performed correlation between the log2(apical/basal) of the two groups (Fig. R4). There is a significant high correlation between both groups:

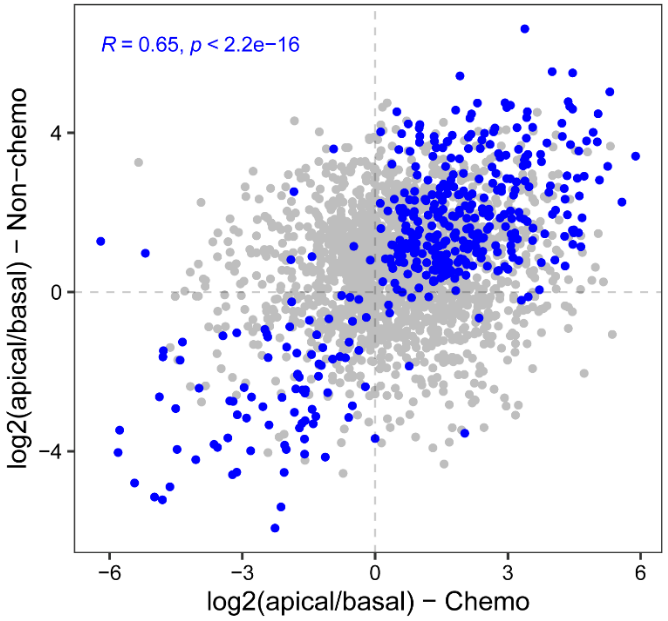

Fig. R4: median log2(apical/basal) of mRNAs in patients that underwent chemotherapy and chemotherapy naïve. Genes with expression > 10^{-5} are shown. significantly polarized genes are in blue. R denotes Spearman's correlation of the significantly polarized genes.

3. The location of SLC5A1 mRNA in mouse being partially basal is one of the surprising observations; please provide the polarity of the protein by IF in mouse intestine. Also calling SLC5A1 discordant for human as in Fig 4D is not correct.

As correctly pointed by the reviewer, we have now changed the caption of Fig. 3D to reflect this (changed "selected discordant genes" to "selected genes"). Indeed, although Slc5a1 mRNA is balanced in mouse, the encoded protein, SGLT1 is strongly apical in mouse, as we have shown in a previous publication from our lab (Fig. R5, Figure 4d in https://www.nature.com/articles/s42255-021-00504-6):

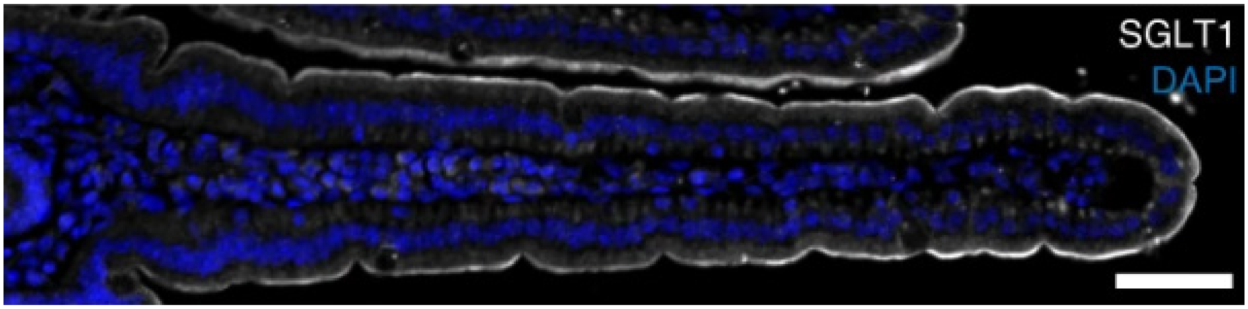

Fig. R5: Immunofluorescence of SGLT1, Scale bar, 50 µm (figure 4d from https://www.nature.com/articles/s42255-021-00504-6).

4. Do not see PNLIP in Fig 4A which would be interesting given that it is thought to sticks apically.

We have now added PNLIP to Fig 4A:

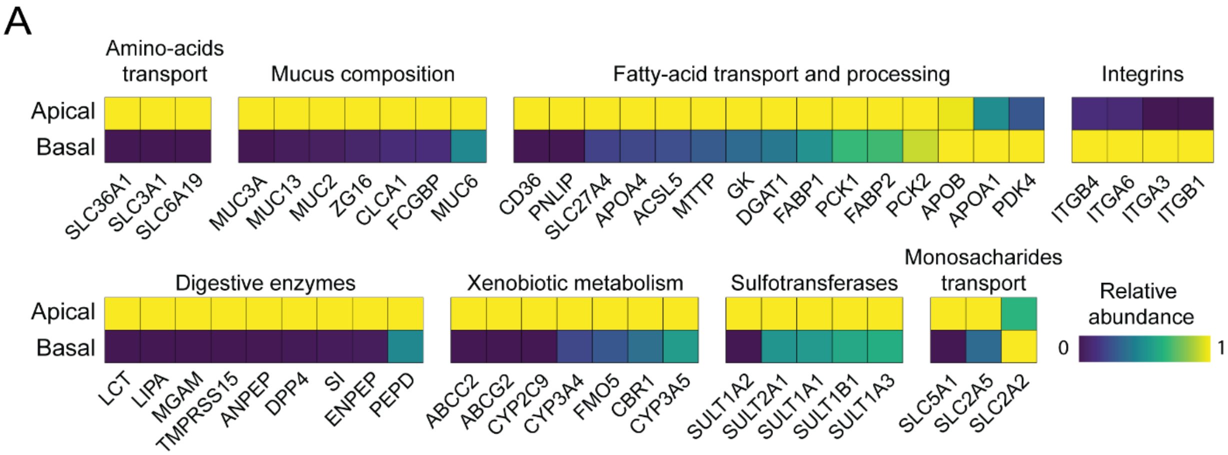

Fig. 4A: Max-normalized abundance of proteins involved in nutrient processing and absorption on the apical and basal sides from LCM-proteomics data.

5. In Methods for RNA-seq formula; explain why pn added (to both numerator and denominator).

We have now clarified this in the methods on page 16:

"pn is pseudo-number which is the minimal normalized expression across all samples and all genes, which is not 0. This value is added to avoid division by zero and consequently infinite or non-defined values when expression levels are low, without skewing the ratios."

Rev. 3:

In this manuscript, Novoselsky and colleagues perform transcriptomics and proteomics analysis of basal and apical portion of small intestinal epithelium from human and mice and validated the findings using single-molecule FISH and immunohistochemistry (obtained from human protein atlas website). They find transcript and proteins with apical or basal localization polarization, which sometimes correlates for the same gene. They suggest presence of a streamlined " nutrient and transport and processing" in intestinal cells. The strength of the paper is the use of human tissues and the comparison with mouse and the systematic approach allowed by using RNA-Seq and Mass Spectrometry. However, there are several issues with the conceptual design of the paper and the methods employed.

1. There is limited usefulness in the assessment of apical/basal localization of mRNA and proteins, especially due to the use of laser-capture microdissections method. Due to the basal localization of the nuclei (also noted by the authors in the results section) and the limited abundance of cytoplasm in the basal side, the use of LCM will result essentially in the comparison of a fraction highly enriched in cytoplasm (the apical part) with a fraction highly enriched in nuclear content (basal side). This is reflected in their data as most of the proteins show apical bias except histones and ribosomes and mitochondria which are known to reside in proximity to the nucleus. Therefore, this analysis is of limited usefulness to really assess cellular localization of proteins. This is also reflected in the limited novelty provided by the paper. This is a crucial point that should be clearly mentioned in the discussion.

We thank the reviewer for the important comment. Indeed, the LCM of subcellular compartments is limited because of the minimal resolution of the laser. Since the basal side of the epithelial cells is rather small, we collected the minimal possible volume that the resolution of the LCM enabled us but tried avoiding the collection of stromal cells as much as possible. This resulted in a small part of the nuclei to be dissected into the basal samples. The RNA-seq protocol has an amplification step via PCR and uses UMIs, further enhancing sensitivity, and thus the data captures the basal genes, as we demonstrated by the smFISH validations (Fig. 2B). In contrast, the proteomics experiment doesn't contain an amplification step, and thus the basal side shows more ECM and nuclear proteins, rather than basally-epithelial proteins.

To address this point, we have now repeated the protein polarization analysis while excluding ECM and nuclear-specific proteins. This revised analysis revealed very high correlation in the apical/basal ratio (Fig. R6A), confirming the intra-cellular polarization of cytoplasmic proteins, and identified 42 proteins with significant basal polarization (Fig. R6B):

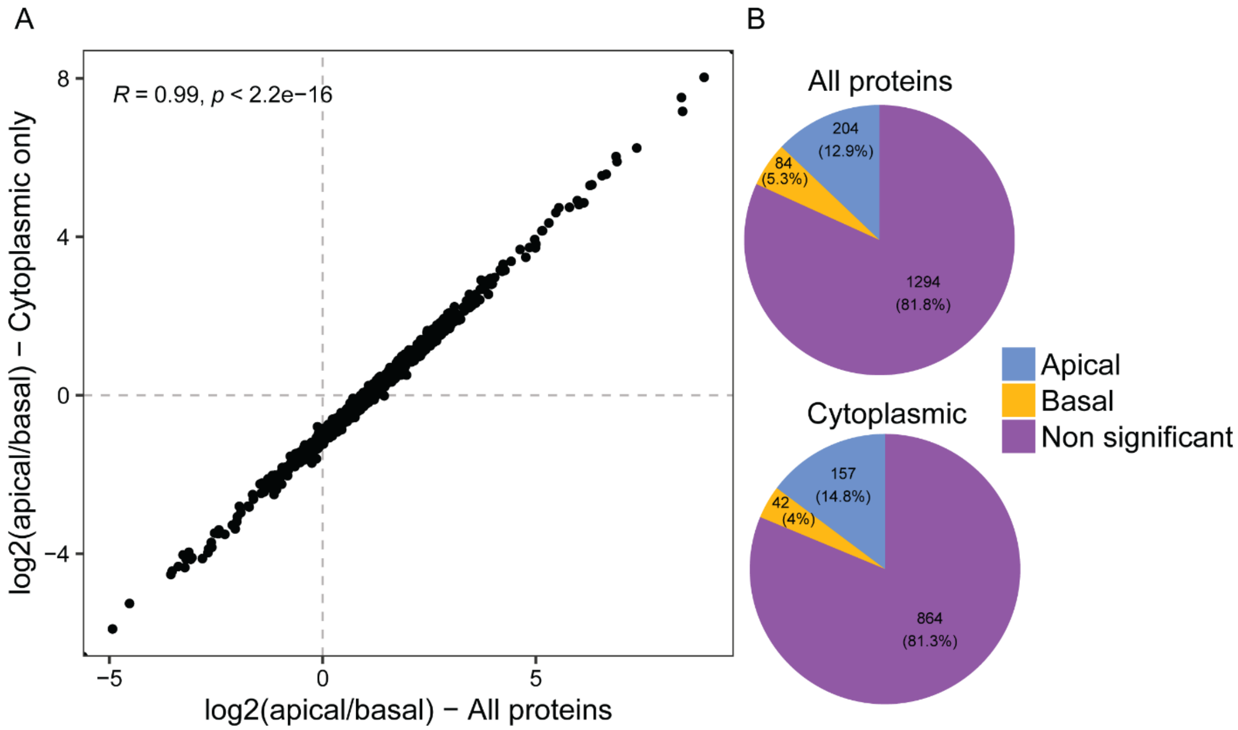

Fig. R6: (A) Scatter plot comparing the log2(apical/basal) ratios of protein polarization between all proteins (x-axis) and cytoplasmic-only proteins (y-axis), with data normalized in both cases. (B) Number of significantly polarized proteins (Deseq2, Q-val < 0.25) between all proteins and cytoplasmic-only proteins, with data normalized in both cases.

We have now added a column in the Supplementary Table 4 that indicates whether a protein is nuclear/ECM to enable refined analysis. Importantly, central conclusions that have emerged from our protein polarization regarding enterocyte function, for example the differential localization of proteins in the lipid digestion and absorption pathway remain unchanged. We now show this in the new Fig S7A:

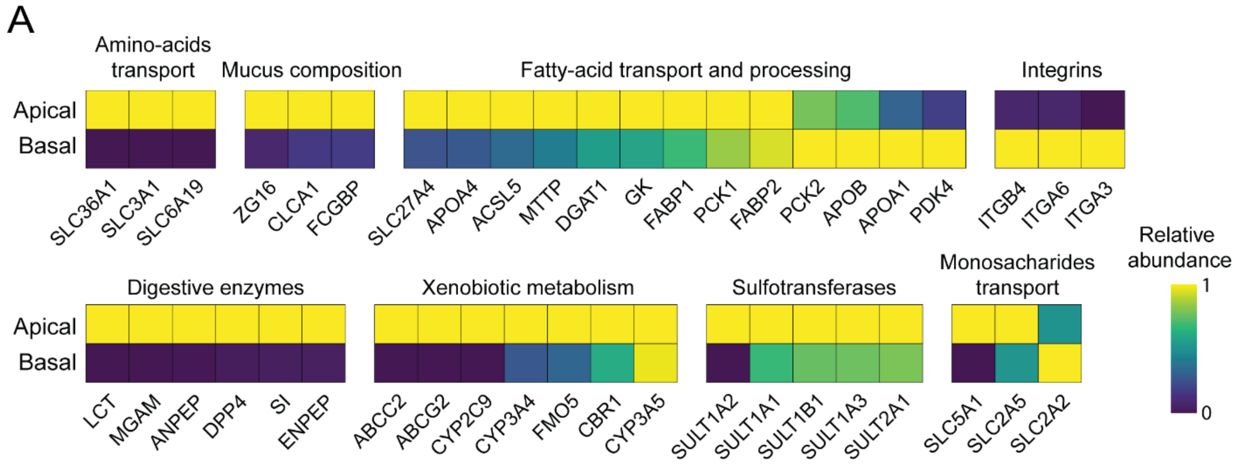

Fig S7A: Max-normalized abundance of proteins involved in nutrient processing and absorption on the apical and basal sides of cytoplasmic proteins, internally normalized, from LCM-proteomics data

And present these results in the text on page 8:

"Notably, these polarization trends remain unchanged after filtering out nuclear and matrix proteins, which are more abundant in the basal samples in our data (Fig. S7A)."

We also now explicitely discuss the differences in sizes between compartments in the Results section on page 3:

"Epithelial segments were carefully dissected to avoid inclusion of stroma. Since the basal compartment in human is significantly smaller compared to the apical compartment (width of 15.4µm vs. 6.6µm, Fig. S1A, Supplementary Table 2), this resulted in generally lower mRNA yields in the basal compartments (Fig. S1B). We therefore normalized the data in each compartment to enable comparison of mRNA and protein concentrations (Methods)."

We show these results in Fig. S1A and Fig. S1B:

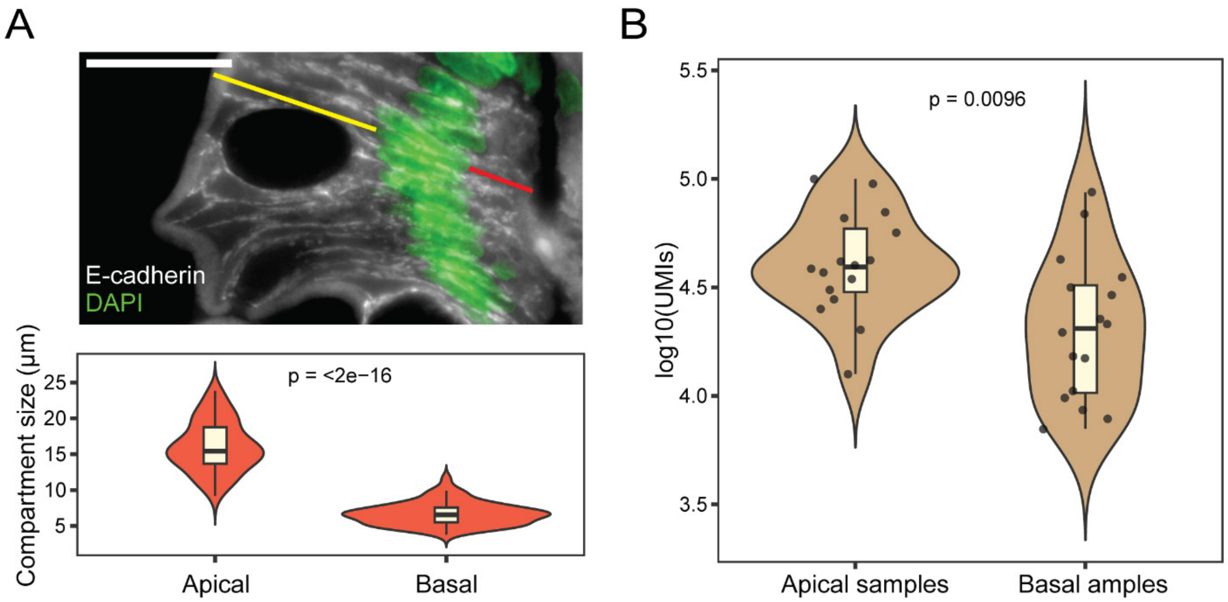

(A) Top – Immuno-fluorescence staining of E-cadherin, used for measurement of apical (yellow) and basal (red) sides of the epithelial cells. Scale bar 20µm. Bottom – quantification of measurements from 8 patients, 3 FOV per patient, 3 measurements per FOV. Boxplots show the medians and 25-75 percentiles, P-value is Wilcoxon rank-sum test. (B) number of unique molecules identifiers (UMIs) across apical and basal samples of LCM RNA-seq of human

proximal jejunums. Boxplots show the medians and 25-75 percentiles, P-value is Wilcoxon rank-sum test.

And detail the associated potential limitation of our study in the Discussion, on page 11:

"The basal compartment in the epithelial cells was at the resolution limit of the LCM, and included residual nuclear fragments and consequently, basal polarization of nuclear proteins (Fig. 4B). Despite this, the main conclusions of the study, including the streamlined polarization of enterocyte-specific proteins remained invariable upon removal of nuclear and stromal proteins (Fig S7A)."

Lastly, we have changed the IHC image of COL6A2 to CASK in Fig. 3A-B, for a better representation of proteins that are highly expressed in epithelial cells, to show that the data does capture basal polarization of the epithelium.

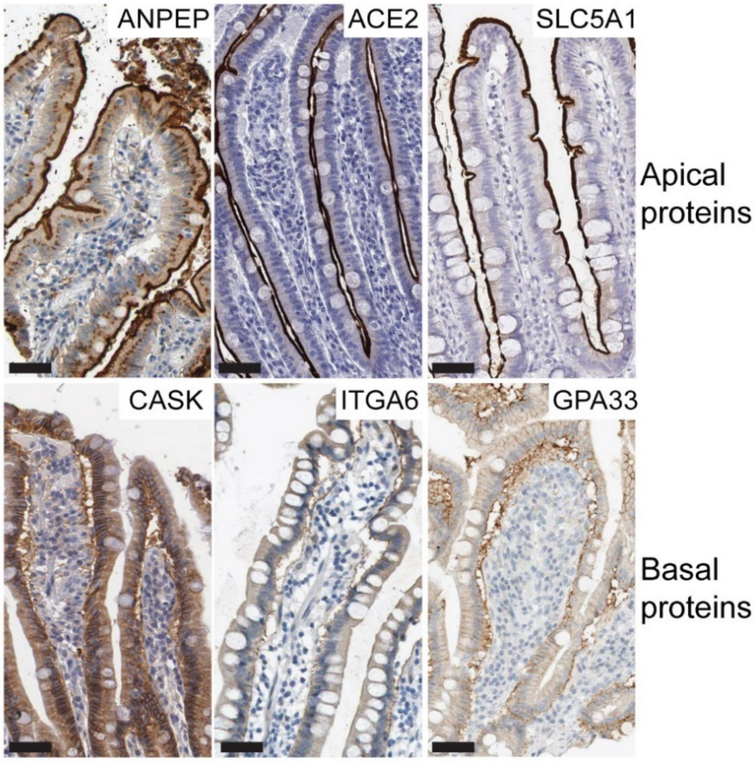

Fig. 3B: Immunohistochemistry (IHC) staining of selected basal (top) and apical (bottom) proteins from the human protein atlas of healthy duodenum or small intestines. Scale bar is 50µm. Direct URLs: ANPEP, ACE2, SLC5A1, CASK, ITGA6, GPA33.

2. In smFISH experiments: there should be noted how many patients were analyzed and how many FOVs were analyzed for each patients. Quantification of the signal in apical VS basal localization should also be carried out. Negative controls should also be shown.

We have now added negative control for smFISH images (Fig. S1E) – which were performed on consecutive slides of a true staining and without probes.

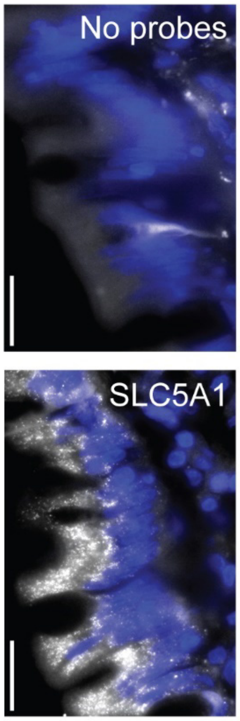

Fig. S1E: smFISH staining of SLC5A1 and a staining without a probe. Scale bar is 20μm.

We have now also performed comprehensive quantification of our smFISH images, measuring the dot intensities of several genes. We found a very strong and significant correlation between the log2(apical/basal) in LCM-RNAseq and in smFISH ($R=0.95$, $p<2.2 \times 10^{-16}$). We have added this analysis to Fig. S1F, and described the number of images analyzed in the figure legend:

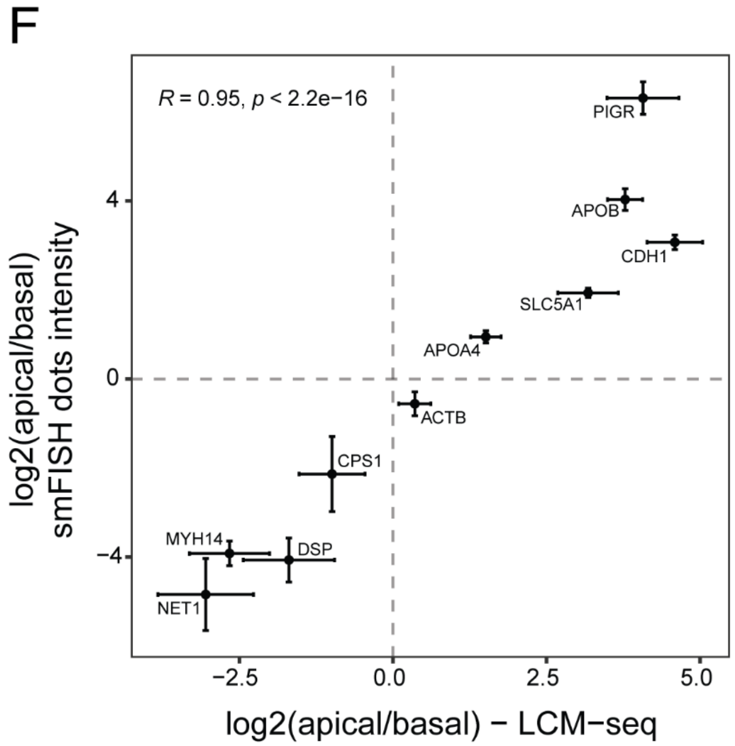

Fig. S1F: Spearman correlation between the log2(apical/basal) in LCM-RNAseq and quantification of dot intensities of smFISH. In smFISH, intensity of dots (normalized to area), from multiple fields of view, from 2-3 patients were quantified. Error bars are standard errors of the means of the log2(apical/basal) from all samples or measurements.

This new analysis is described in the Methods section on page 16:

"smFISH images were analyzed by manual segmentation of apical and basal compartments of few cells, and identification of dots using imageM (32). For each gene, multiple FOVs from distinct 3 villi per patient, across 2-3 patients were quantified. Spot intensity was normalized by the segmented area and the log2(apical/basal) ratio was calculated for each paired segmentation."

3. It seems that there are multiple repeats of the same image: -CDH1 staining from Fig 3D is a repeat of 2B (image rotated), SLC5A 2A is a repeat of 2D and 3D, this should be replaced with different representative images.

We have now replaced the images of Slc5a1 in Fig 2D and Fig 3B, and Myh14, Cdh1 in Fig. 3B with different representative images.

4. In figure 2D: ApoB FISH is also shown but not mentioned in the text, therefore the logic of showing this is unclear.

We have now added a mention in the main text on page 4:

"We validated two of these genes with smFISH – PIGR was significantly apical in humans yet significantly basal in mice, whereas SLC5A1 was significantly apical in humans yet non-polarized in mice. We also validated APOB, which shows apical polarization in both species (Fig. 2D)."

5. Data in figure 4a should also been show as box plots, histograms or violin plot showing single-point data and error bars.

We have added Fig. S4, which consists of boxplots containing the medians and 25-75 percentiles for each of the genes shown in Fig. 4A:

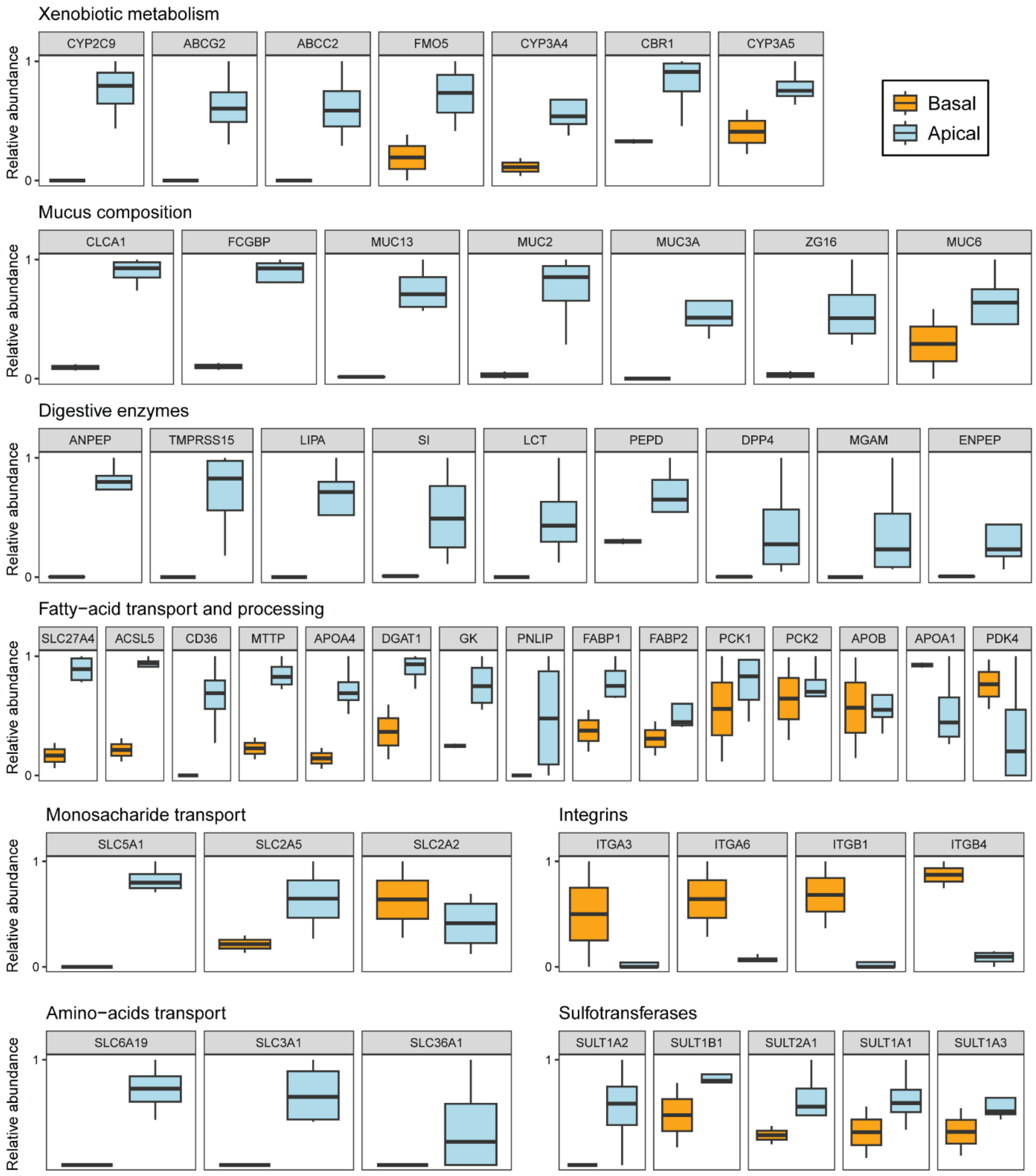

Fig S4: Max-normalized abundance of proteins involved in nutrient processing and absorption on the apical and basal sides from LCM-proteomics data. Horizontal bars are medians, boxes delineate the 25-75 percentiles.

6. Number of patients analyzed for each figure/panel should also be reported in the legends for ease.

We added the number of patients in the figure's legends Fig. 2A, Fig. 3A, Fig. S1A, Fig. S1F.

7. When handful of genes are highlighted (ie Fig2C, 3A and C) it should be specified that these are selected by authors not statistically.

We have added a clarification in the figure's legends Fig. 2A, Fig. 2C, Fig. 3A, Fig. 3C.

8. The concept of apicome is not explained clearly and generally confusing. In line 72 for instance it would read more clearly: "the samples clustered by their apical or basal origin and villi zone rather than…" is the apicome only apical protein? Is the comparison of apical and basal? The term should be changed or use it differently in the text.

We have now changed the sentence as suggested by the reviewer:

"The samples clustered by the apical/basal origin and villi zones, rather than by patient (Fig. S1C)."

We have now also clarified the definition of "apicome" on page 2:

"Here we used LCM to assemble the ratios between apical and basal mRNAs and proteins – henceforth termed "apicome", of the human and mouse small intestines."

9. It would also be useful to expand on the "apicome score" for instance by indicate that positive numbers imply apical polarization and negative number a basal polarization. Also, in figure this is expressed as log2(apical/basal) rather than with the term "apicome score", perhaps unifying the terminology would make manuscript clearer.

We have changed all occurrences of "apicome score" in the manuscript to "log2(apical/basal)" for clearance.
